# Development and evaluation of a risk assessment tool to improve clinical triage accuracy for colonoscopic investigations

**DOI:** 10.1186/s12885-018-4140-0

**Published:** 2018-02-27

**Authors:** Anton R. Lord, Lisa A. Simms, Allison Brown, Katherine Hanigan, Krupa Krishnaprasad, Belinda Schouten, Anthony R. Croft, Mark N. Appleyard, Graham L. Radford-Smith

**Affiliations:** 1 Inflammatory Bowel Diseases, QIMR Berghofer Medical Research Institute, Brisbane, Australia; 2 Department of Gastroenterology and Hepatology, Royal Brisbane and Women’s Hospital, Brisbane, Australia; 3 University of Queensland School of Medicine, Brisbane, Australia

**Keywords:** Risk assessment tool, Colorectal cancer, Colonoscopy, Bowel disease, Faecal immunochemical test

## Abstract

**Background:**

Gastroenterology Departments at hospitals within Australia receive thousands of General Practitioner (GP)-referral letters for gastrointestinal investigations every month. Many of these requests are for colonoscopy. This study aims to evaluate the performance of the current symptoms-based triage system compared to a novel risk score using objective markers.

**Methods:**

Patients with lower abdominal symptoms referred by their GPs and triaged by a Gastroenterology consultant to a colonoscopy consent clinic were recruited into the study. A risk assessment tool (RAT) was developed using objective data (clinical, demographic, pathology (stool test, FIT), standard blood tests and colonoscopy outcome). Colonoscopy and histology results were scored and then stratified as either significant bowel disease (SBD) or non-significant bowel disease (non-SBD).

**Results:**

Of the 467 patients in our study, 45.1% were male, the mean age was 54.3 ± 13.8 years and mean BMI was 27.8 ± 6.2. Overall, 26% had SBD compared to 74% with non-SBD (42% of the cohort had a normal colonoscopy). Increasing severity of referral symptoms was related to a higher triage category, (rectal bleeding, *P* = 2.86*10^-9^; diarrhoea, *P* = 0.026; abdominal pain, *P* = 5.67*10^-4^). However, there was no significant difference in the prevalence of rectal bleeding (*P* = 0.991) or diarrhoea (*P* = 0.843) for SBD. Abdominal pain significantly reduced the risk of SBD (*P* = 0.0344, OR = 0.52, CI = 0.27-0.95). Conversely, the RAT had a very high specificity of 98% with PPV and NPV of SBD prediction, 74% and 77%, respectively. The RAT provided an odds ratio (OR) of 9.0, 95%CI 4.29-18.75, *p* = 2.32*10^-11^), higher than the FIT test (OR = 5.3, 95%CI 2.44-11.69, *p* = 4.88*10^-6^), blood score (OR = 2.8, 95%CI 1.72- 4.38, *p* = 1.47*10^-5^) or age (OR = 2.5, 95%CI 1.61-4.00, 5.12*10^-5^) independently. Notably, the ORs of these individual objective measures were higher than the current practice of symptoms-based triaging (OR = 1.4, 95%CI 0.88-2.11, *p* = 0.153).

**Conclusions:**

It is critical that individuals with high risk of having SBD are triaged to the appropriate category with the shortest wait time. Here we provide evidence that a combination of blood markers, demographic markers and the FIT test have a higher diagnostic accuracy for SBD than FIT alone.

## Background

The gastrointestinal tract is the largest epithelial surface in the human body, and hence represents a major source of both pre-malignancy and malignancy [1]. In Australia, bowel cancer screening commenced in 2008, securing ongoing funding for this initiative in July 2011. One million Australians who turned 50, 55 and 65 between 1st January 2011 and 31st December 2014 were invited to participate in this programme [2, 3]. However, individuals in this programme who record a positive faecal immunochemical test (FIT) represent only a small fraction (4.3%) of colonoscopy services undertaken within Australia each year [4, 5]. The majority of colonoscopy services in Australia, including public and private healthcare sectors, are undertaken in the symptomatic population (subjects referred by primary care doctors with a range of symptoms referrable to the gastrointestinal tract) [6]. The increasing public and clinician awareness of both colorectal and upper gastrointestinal malignancies, together with the increasing prevalence of non-malignant (inflammatory bowel disease (IBD), gastro-oesophageal reflux disease), functional gastrointestinal disorders and surveillance colonoscopies has led to an enormous increase in the demand for gastrointestinal endoscopic services [4, 7].

Symptoms alone are a poor indicator of significant bowel disease (SBD) such as cancer, high risk adenoma, and IBD [8]. Australian prospective data on symptom prediction for bowel cancer include a NSW study which recruited over 8000 patients from secondary care after GP referral. Symptoms did not add significantly to the performance characteristics yielded by age and other components within medical history [6]. The investigators developed a multivariate model aimed at discriminating between those at high and low risk of bowel cancer. This work also estimated that up to 95% of bowel cancers could have been detected with only 60% of the colonoscopies performed during the study period. Thus, in the absence of adequate clinical data available at the time of triage, a large proportion of patients are undergoing an invasive procedure that may not be clinically justified based on pre-test risk of SBD.

Several systematic reviews demonstrate a wide range in performance characteristics for a number of symptoms and groups of symptoms as predictors for colorectal cancer. Sensitivities range between 0.13 for iron deficiency anaemia and 0.44 for rectal bleeding, with specificity at 0.92 and 0.66 respectively [9]. To date, only the FIT has demonstrated clinically reasonable sensitivity (0.95) and specificity (0.84) [9].

Tools that can accurately predict SBD in the symptomatic population have potential advantages over those that predict bowel cancer alone. Patients identified at an earlier stage of their disease (high-risk adenoma, early stage IBD) have better outcomes compared to those diagnosed with later stage disease [10, 11]. However, currently available risk assessment tools (RATs) focus on bowel cancer as their primary outcome [12, 13].

A cut-off value of 10 μg/g faecal haemoglobin is commonly used in bowel cancer screening programs in asymptomatic populations. While the appropriate FIT cut-off for faecal haemoglobin in the symptomatic population is yet to be determined, data from two recent studies [14, 15] indicate that a cut-off of ‘any detectable faecal haemoglobin’ could be a reliable ‘rule out’ test for significant bowel pathology (bowel cancer, advanced adenomas, IBD), regardless of gender, with a negative predictive value of over 97% [15]. Based upon these data together with FIT metrics for predicting colorectal cancer [9], this test alone or as part of a RAT may prove to be more clinically effective and safer for triaging symptomatic patients.

The aim of our study was to evaluate the accuracy of the symptoms-based triage system compared to a RAT developed using objective markers only (FIT ± blood test/s ± demographic data) for identifying or ruling out significant bowel pathology. Suboptimal triaging places a large burden on Gastroenterology departments. Moreover, a 12-month delay in screening for patients with SBD means that an estimated 2.6 to 5.6% of patients with SBD will transition to colorectal cancer while waiting for the colonoscopy [16], significantly altering available therapeutic strategies and clinical outcomes. Prioritising patients with a pre-test high risk of SBD will reduce the time until diagnosis and enable the early implementation of therapeutic interventions.

## Methods

### Participants

The study cohort was recruited through one of three weekly colonoscopy consent clinics and comprised GP-referred patients within the Brisbane Metro North division (catchment area of Royal Brisbane and Women’s Hospital (RBWH)) requiring colonoscopic investigation for lower abdominal symptoms. Study exclusion criteria included age below 20 years and over 85 years, and patients who after reading the study education and consent form declined participation. Consented participants in the study provided a stool sample (last bowel movement prior to taking their colonoscopy preparation), and had a blood sample taken on the day of their procedure for standard blood tests.

### Data collected

The following data items were collected; objective demographic data (age, gender, BMI); blood and stool tests (full blood count, C-reactive protein, serum biochemistry (Chem20), iron studies, lipid profile, faecal calprotectin and faecal haemoglobin (iFOB or FIT), referral information including referral indication, dates of referral, triage date and triage category; attached GP-requested investigations (blood, stool, radiology, scans); number of bowel symptom-related GP visits 6 months prior to colonoscopy referral date; family history of colorectal cancer; smoking and alcohol history; date of colonoscopy and findings; histology results; and study withdrawal reasons if applicable. Blood, stool and colonoscopic biopsies were processed by Pathology Queensland and Envoi Specialist Pathologists. Histology reports were reviewed by a single gastroenterologist (GLR-S), scored and stratified for analysis into two groups, SBD or non-SBD. SBD was defined as any presence of cancer, or high risk adenomas (adenomas ≥10 mm, ≥3 adenomas at procedure, or presence of villous component or high-grade dysplasia) [15, 17]. Non-SBD was classified as any adenomas < 10 mm, < 3 adenomas at procedure, hyperplastic polyps, or presence of no dysplasia/low-grade dysplasia only, other e.g. non-specific focal inflammation related to the bowel preparation, normal colonoscopy.

### Statistical analyses

Continuous objective markers such as blood or stool markers, age, BMI etc. were categorised into ordinal data prior to assessing statistical significance. Age was categorised into older or younger than 65 years, BMI into underweight (< 18), normal (18-24), overweight (25-29), obese (30-39) and morbidly obese (> 40), pathological tests were categorised into low, normal or high based on individual reference ranges. The impact of binary categorical variables on the presence of SBD was calculated using the odds ratio (OR). Odds ratios for blood and stool markers were calculated by considering a high or low level independently of the particular marker compared to a normal reading. The impact of factors on triage category was calculated using a generalised linear model, where the reference group was category 3 (least urgent category).

Blood markers used in the generation of the blood risk score (BRS) were identified through mass univariate OR testing. Blood tests with a significant OR (*P* < 0.05, false discovery rate (FDR) corrected) were combined into the BRS. Each abnormal test (high for triglycerides (reference range: > 1.5) or glucose (reference range: 3.0-7.8); low for magnesium (reference range: 0.7-1.1) or creatinine (reference range: 64-108) added one point to the score, up to a maximum of 4 points.

**Fig. 1:**
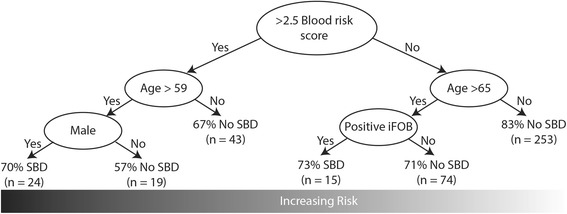
Algorithm for calculating the risk of SBD prior to colonoscopy

An algorithm for risk assessment was created using the combined blood risk score, age and faecal haemoglobin test (Fig. 1). The algorithm was assessed using positive and negative predictive values. The OR of predicted outcomes based on the algorithm was compared against current best practice. To ensure a fair comparison triage categories were refactored into high risk (category 1) and low risk (category 2/3). For the OR calculations, exposure was defined as the RAT predicting SBD or assignment to triage category 1. Disease was defined as the presence of SBD as outlined above. All analyses was performed in R using the ‘fmrb’ and ‘rpart’ packages.

## Results

### Study population

A total of 467/663 patients recruited into the study had completed their colonoscopy procedure at the time of analysis. 214/467 (45.1%) participants were male and mean age (range) was 54.3 ± 13.8 years with 33.2% under 50 years of age (Table 1). Mean (standard deviation) BMI was 27.8 ± 6.2. Family history of colorectal cancer affecting one or two first-degree relatives diagnosed > 50 years was reported by 71/250 (28.4%) patients. Regarding lifestyle factors, 19.6% of patients in our study were smokers, 33.3% were ex-smokers with 47.0% having never smoked. Seventy-four percent of our study population identified as current drinkers, together with 14.9% as ex-drinkers and 11.1% as non-drinkers. Diabetes was present in 12.4% of the study cohort.

**Table 1:** Demographic and clinical characteristics of the study population

Demographic/Clinical history	*n* (%)
Age in years, *n* = 467	
< 50	151 (32.3%)
Gender, n = 467	
Male	214 (45.1%)
Body Mass Index, BMI	27.8 ± 6.2
Diabetes, *n* = 451	56 (12.4%)
History of colorectal cancer, *n* = 418^a^	177 (42.3%)
Smoking status, *n* = 461	
Never	221 (47.9%)
Current	90 (19.5%)
Ex-smoker	150 (32.5%)
Alcohol history, *n* = 446	
Non-drinker	59 (13.2%)
Current drinker	319 (71.5%)
Ex-drinker	68 (15.2%)
General Practitioner consultation	
GP-requested blood test prior to colonoscopy, *n* = 451	276 (59.1%)
GP-requested stool tests (iFOB or FIT) prior to colonoscopy, *n* = 451	90 (19.3%)
Any GP-requested scan/radiology, *n* = 451	55 (12.2%)
Number of bowel symptom-related GP visits 6 months prior to referral date, *n* = 445	
0	190 (42.7%)
1	166 (37.3%)
2	54 (12.1%)
> 2	35 (7.9%)

^a^first or second-degree relative

Of the 467 patients with lower abdominal symptoms, 44.1% of referred patients had undergone clinically useful tests (blood and/or stool) prior to their referral including a full blood count and ferritin (iron deficiency anaemia), with only 20% having had a faecal haemoglobin test (iFOB or FIT). Eighty-nine (19.1%) of patients had visited their GP (in the 6 months prior to their referral date) on two or more occasions for bowel symptom-related consultations.

**Fig. 2:**
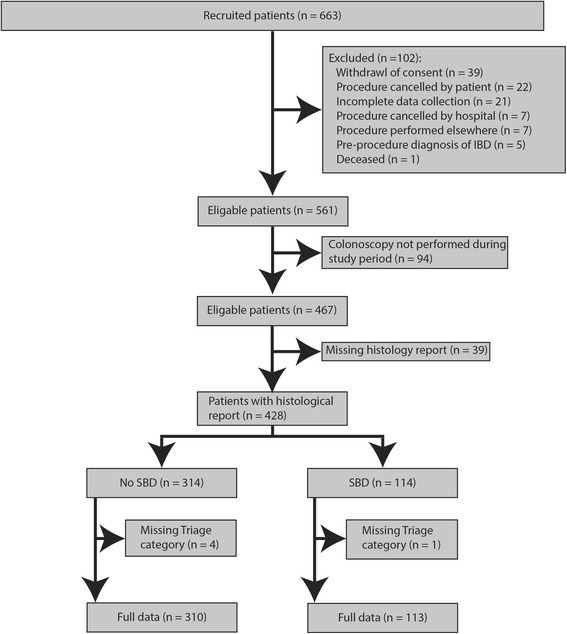
STARD showing study population inclusion and available data

A subset of patients, 102/663 (15.4%), withdrew from the study for a variety of reasons including consent withdrawal, *n* = 39 (38.2%); procedure cancelled by patient, *n* = 22 (21.6%); incomplete data collection, *n* = 21 (20.6%); procedure cancelled by hospital, *n* = 7 (6.9%); procedure performed elsewhere, *n* = 7 (6.9%); pre-procedure diagnosis of IBD, *n* = 5 (4.8%); and patient deceased, *n* = 1 (1.0%) (Fig. 2).

### Referral characteristics and colonoscopy waiting times

All GP-referral letters were triaged by gastroenterology consultants to the appropriate procedure(s) and classified as either category 1, 2 or 3 based on procedure urgency, with category 1 being most urgent. The most frequent GP-reported symptom was altered bowel habit (36.3%) followed by rectal bleeding (34.7%), abdominal pain (17.6%), iron deficiency anaemia (16.2%) and a positive iFOB/FIT test (8.3%). We observed no relationship between SBD and altered bowel habit (*P* = 0.641), rectal bleeding (*P* = 0.991), abdominal pain (*P* = 0.58). Iron deficiency anaemia was observed to be protective from SBD (*P* = 0.015, OR = 0.42 CI:0.21- 0.86), however, this effect was driven by the female population only (female: *P* = 0.055, OR = 0.39, CI:0.14-1.05; male: *P* = 0.37, OR = 0.62, CI:0.21-1.78). Within the female population, women under 50 showed a stronger effect (*P* = 0.134, OR = 0.36, CI:0.10-1.41) compared to those over 50 (*P* = 0.407, OR = 0.52, CI:0.11-2.46).

Examining the distribution of symptoms between patients with and without SBD, we found no significant difference between referral symptoms and assigned triage category, (altered bowel habit, *P* = 0.27; rectal bleeding, *P* = 0.51; diarrhoea, *P* = 0.91; abdominal pain, *P* = 0.062).

**Fig. 3:**
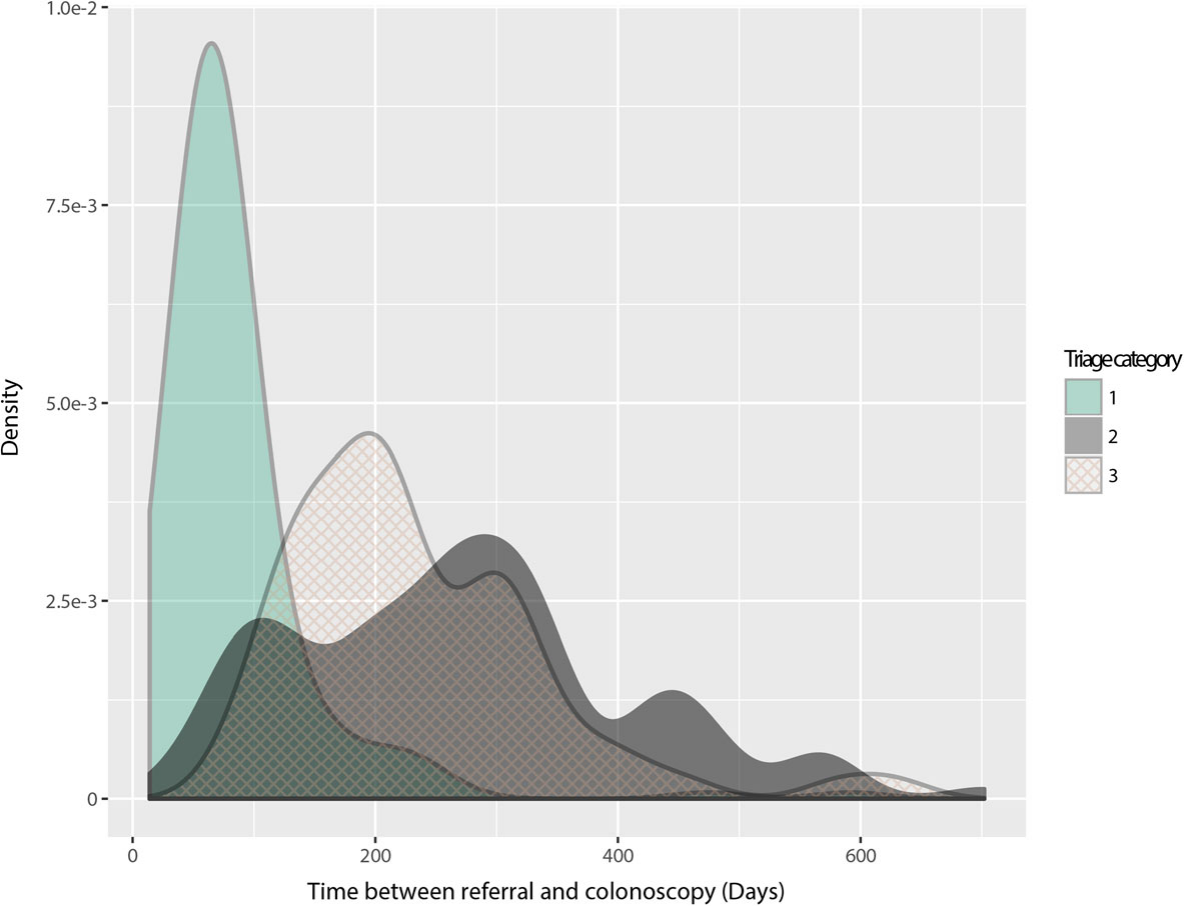
Time between referral and colonoscopy for high-, medium- and low-risk patients (category 1, 2 and 3, respectively)

The recommended waiting times for category 1, 2, and 3 are 1 month, 3 months and 12 months, respectively. In our study population, the median waiting times between the initial referral from a GP until colonoscopy for category 1, 2 and 3 were 69 days (IQR, 53-86.5 days), 264 days (IQR, 175-331.5 days), and 220 days (range, 179.5-309 days), respectively (Table 2, Fig. 3).

**Table 2:** Median colonoscopy waiting times for symptomatic patients in days (IQR) by triage category

Category	*n*	GP-referral to triage	Triage to clinic appointment	Clinic appointment to colonoscopy
1	212	6 (1-12)	27 (17-38)	34 (25-46)
2	159	6 (2-12)	84.5 (56-116.2)	142.5 (91.5-136.2)
3	90	5 (0-12)	83 (30-142)	124 (89.25-153)

No difference in waiting time between GP referral and triage was observed (median 5 days, IQR 0-12 days). Category 1 patients attended the colonoscopy consent clinic (CCC) significantly earlier than category 2 or category 3 patients with a median waiting time (IQR) between triage and CCC visit of 34.5 days (24-50), 98.5 days (66.5-132.2) and 78 days (62.75-135), respectively. The median waiting time (IQR) between CCC visit and colonoscopy procedure was 35 days (26-47), 133.5 days (89.75-234.2) and 127 days (91-167) for category 1, 2 and 3, respectively. A post-hoc analysis on the waiting time between CCC appointment and colonoscopy revealed category 1 patients were seen 136 days earlier than category 2 patients and 102 days earlier than category 3 patients (*P* < 0.0001 for both). Interestingly, the waiting time between CCC visit and colonoscopy procedure was 32 days shorter for category 3 patients compared to category 2 patients (*P* = 0.006). Notably, the number of patients assigned to category 2 was much greater than that assigned to category 3 (Table 2).

### Colonoscopy findings

Of the 428 patients with histological findings available, colorectal cancer was identified in 5 (1.2%), and high-risk adenomas were found in 107 (25.0%). Of these 107 patients, 34 (31.8%) were identified as high risk due to large or multiple serrated polyps. Overall, 26% patients had SBD compared to 74% with non-SBD. One hundred and ninety-seven patients (46.0%) had a normal colonoscopy, that is, no polyps, adenomas, cancer, or IBD. There was no significant difference in the prevalence of symptoms in patients with and without SBD (altered bowel habit, *P* = 0.770; rectal bleeding, *P* = 0.992; diarrhoea, *P* = 0.842, and abdominal pain, *P* = 0.080).

### Risk assessment tool development

**Fig. 4:**
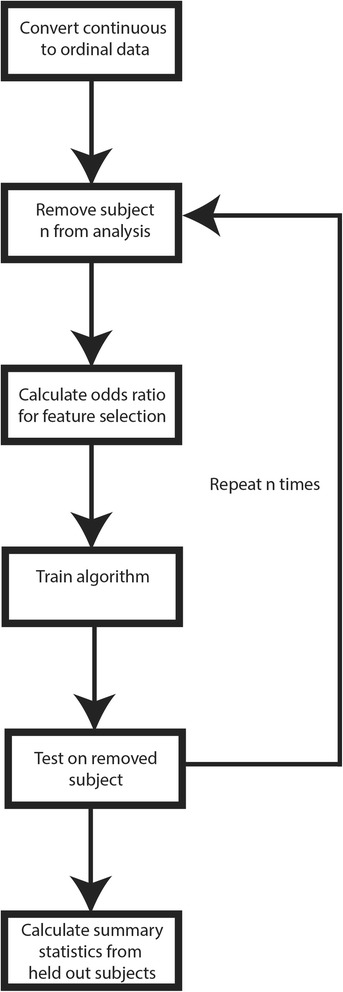
Flowchart detailing leave one out cross validation from the RAT

Markers used in the risk assessment tool (RAT) were selected through mass univariate OR testing. That is, the OR was calculated for each objective marker independently. The *P*-values for each marker were corrected for the number of tests performed using the FDR [18]. Markers identified with a FDR corrected *P*-value < 0.05 were considered statistically significant. Leave one out cross validation was performed on feature selection and training/testing the algorithm for risk assessment (Fig. 4). As such, one sample was removed from the analysis and the odds ratios were then calculated. Measures with a significant OR were used as features and the algorithm was trained on all data minus the left out sample. A prediction of SBD was derived for the final sample using the algorithm for risk assessment and the predicted outcome was compared against the histology reports for that sample. This procedure was repeated until each sample had been left out once. Predicted versus confirmed SBD for the entire dataset was then used to calculate a confusion matrix, from which sensitivity, specificity and the OR of the algorithm for risk assessment were derived.

### Symptoms versus risk assessment tool

Individual referral symptoms showed almost no predictive power for the identification of SBD with only a positive FIT test identified as a risk factor (Table 3). Notably, of the 30 patients with large and/or three or more serrated polyps who also had undertaken the FIT test, only two (6.7%) returned a positive result.

**Table 3:** Performance of referral symptoms and FIT in identifying significant bowel pathology

Referral symptom	SBD *n* = 114 Positive n (%)	No SBD *n* = 314 Positive n (%)	OR	95%CI	*P*-value
Altered bowel habit	40 (38.5)	115 (36.6)	0.93	0.60-1.46	0.770
Rectal bleeding	40 (38.5)	110 (35)	1.02	0.64-1.57	0.992
Diarrhoea	36 (31.6)	96 (30.5)	1.04	0.66-1.66	0.842
Abdominal pain	13 (11.4)	61 (19.4)	0.56	0.30-1.08	0.080
Iron deficiency anaemia	10 (8.7)	256 (81.5)	0.44	0.21-0.86	0.015
Positive faecal haemoglobin	19 (16.6)	20 (6.4)	2.94	1.51-5.74	0.001

Patients predicted as having SBD by the algorithm for risk assessment had an increased risk of SBD confirmed after histology (OR = 9.0, 95%CI 4.29-18.75, *P* = 2.32*10^− 11^). The independent factors comprising the algorithm for risk assessment; the FIT test (OR = 5.3, 95%CI 2.44-11.69, *P* = 4.88*10^− 6^), BRS (OR = 2.8, 95%CI 1.72- 4.38, *P* = 1.47*10^− 5^) and age (OR = 2.5, 95%CI 1.61-4.00, *P* = 5.12*10^− 5^) were also predictive, however to a lesser extent. Faecal calprotectin was also a risk factor (OR = 1.8, 95%CI 1.07-2.95, *P* = 0.024), however its inclusion in the RAT did not improve the overall accuracy and as such it was excluded.

Increases in the BRS were accompanied by an increase in the percentage of the population with SBD. Fifty three out of 255 (20.7%) of the population with a BRS of 1 had SBD, increasing to 45/137 (32.8%), 14/34 (41.2%) and 2/2 (100%) for BRS values of 2, 3 and 4, respectively. The BRS was not related to the current triage system with 114/251 (45%) of category 1 patients having a BRS of 1 and 69/124 (55.6%), 19/33 (57.6%) and 2/2 (100%) having a BRS of 2, 3 or 4, respectively. Notably, the RAT outperformed current triaging practices (OR = 9.0 vs. 1.4, Table 4), which had little informative value for identifying patients with SBD prior to colonoscopy.

**Table 4:** Accuracy and distribution of assigned colonoscopy waiting list categories using GP-reported symptoms compared to risk assessment tool

	SBD, Positive *n* (%)	No SBD, Positive n (%)	OR	95% CI	*P*-value
Triage category classification	*n* = 113^c^	*n* = 310^c^			
Symptoms-based^b^			1.4	0.88-2.11	0.153
Category 1	61 (54.0%)	143 (46.1%)			
Category 2/3	52 (46.0%)	167 (53.8%)			
Risk Assessment Tool	*n* = 114	*n* = 314	9.0	4.29-18.75	2.32^a^10^−11^
Category 1	28 (24.6%)	11 (3.5%)			
Category 2/3	86 (75.4%)	303 (96.5%)			

^a^Category 1 (most urgent), Category 2 (moderately urgent), Category 3 (least urgent)

^b^Current practice

^c^5 patients have been excluded due to missing triage category information. The denominators for percentages are based on the total amount of available data

## Discussion

Presently, in the majority of gastroenterology practices, GP-referrals for colonoscopy are triaged into prioritisation categories based predominantly on reported patient symptoms such as rectal bleeding and abdominal pain. Several studies report that lower abdominal symptoms are a poor guide for identifying patients with significant bowel pathology [6, 8, 9]. We describe here the development of a risk assessment tool as part of a colonoscopy referral management strategy to improve the rate of detection for significant bowel pathology.

In total, complete data sets (blood, stool, colonoscopy and histology results) were available from 428 GP-referred patients. Our results show that the frequency of commonly reported symptoms such as altered bowel habit, rectal bleeding, diarrhoea and abdominal pain were not significantly different between those patients with and without findings of SBD. These results are in agreement with previous studies [8, 15]. In our cohort, 1.1% had colorectal cancer, 26% had SBD (CRC, high-risk adenomas or IBD), and 42% had a normal colonoscopy (no polyps, adenomas, cancer or IBD). The low rate of colorectal cancer in our symptomatic population presenting for colonoscopic investigation is in agreement with others [6, 15, 19]. Half of the study referred patients had undergone clinically useful tests prior to their referral including a full blood count and ferritin, with only 20% having had a faecal haemoglobin test. Many of these tests were requested by the triaging consultant at RBWH. However, this was sporadic, operator dependent and not based upon protocol.

The pathology tests assessed for inclusion in the RAT was based on blood and stool tests collected on the day of colonoscopy instead of at referral. This was due to the fact that there were many tests not performed by GPs at the time of referral; hence we used the more consistently obtained markers taken during the hospital admission.

The diagnostic accuracy of a faecal haemoglobin test for detection of significant colorectal disease in a symptomatic population has considerable value; particularly as a rule-out test for CRC given its high NPV and sensitivity when the cut-off is set at any detectable faecal haemoglobin (CRC: NPV 100%, sensitivity 100%; SBD: NPV = 96.2%, sensitivity = 88.2%) [15]. However, the FIT test is not the optimal rule-in test given its low PPV with many false positives being detected [14, 15, 19]. Furthermore, a substantial minority (31.8%) of SBD in our study were identified based on the size and/or number of serrated polyps. The FIT test only captured 6.7% of these, highlighting the need for other objective markers to complement the FIT given that 22.1% and 15.8% of patients with SBD defined by the size and number of serrated polyps, respectively, develop colorectal cancer [20]. As part of our study protocol, recruited patients were requested to provide a stool sample (last stool prior to taking the colonoscopy preparation), and on the day of the procedure, collection of a blood sample for standard blood tests. Applying the faecal haemoglobin test alone (any detectable faecal haemoglobin) improved the diagnostic accuracy for SBD detection in our cohort, OR 5.3, versus OR 1.4 for symptoms only.

With additional blood and objective demographic markers (age, gender, BMI), we have further improved the performance characteristics of the model (RAT OR = 9.0: FIT alone, OR = 5.3; reported symptoms, OR = 1.4). Adelstein et al. [6] developed a RAT for colorectal cancer based on symptoms, disease history and demographic information. As in the Adelstein study [6], we also identified age, gender and rectal bleeding (identified through the FIT) as risk factors, and that symptoms added little value over other markers. Anaemia has previously been identified as a risk factor for colorectal cancer [6], however we found iron deficiency anaemia to be protective in a female population. Given our cohort is predominantly cancer free (98.9%), the absence of iron deficiency anaemia as a risk factor suggests it is specifically related to colorectal cancer. Since the protective effect was observed in the female population only and appears to be stronger in pre-menopausal women, this suggests an underlying gender specific biological process was involved.

The improvement in performance over the FIT test alone is due to the combination of multiple factors, which allow the tool to identify more homogeneous sub-populations based on age, gender, FIT and blood marker profiles. Notably, unlike the FIT test, the RAT has a high PPV making it capable of identifying a subset of patients with SBD accurately. This allows for an objective, rule based triaging of patients meeting these criteria to be prioritised for urgent assessment. Due to the objective nature of the RAT it could conceivably be used by support staff within the hospital setting, reducing the burden on specialists. The RAT in its current form captures approximately 24.5% of all SBD patients in our study population whereas the category 1 classification using current triage methods capture 53.9%. Notably, the PPV of those identified at high risk by the RAT was 74%, compared to 30% for the current best practice. Given that PPV for patients captured by the RAT was superior to current best practice, this tool could be used to identify a subset of patients who are at an increased risk of SBD. Capturing other patients with SBD would still require professionals trained in identifying patients at the highest risk through traditional means. As such, the RAT will not replace current triage practices; rather it could be used as an initial test to identify an extremely high risk group. Future work will endeavour to expand the data used to fine tune the tool to improve the PPV of the RAT. However, the RAT currently outperforms best practice and shows value if incorporated into the current triaging system.

The increasing demand for colonoscopy services is placing significant pressure on colonoscopy resources in both the public and private healthcare environment. The recommended waiting times for colonoscopy following the initial GP consultation for triaged categories 1 (most urgent), 2 (moderately urgent), and 3 (least urgent) are 1 month, 3 months and 12 months, respectively. In our study population, the median waiting times between the initial referral from a GP until colonoscopy for category 1 was 69 days (IQR, 53-86.5 days). Interestingly, in our cohort, patients triaged to category 3 were seen on average earlier than category 2 patients (220 days (range, 179.5-309 days), and 264 days (IQR, 175-331.5 days, respectively. These findings highlight the workload pressures on a major referral centre and demonstrate a service where demand is outstripping resources. The key issue is prioritisation of patients within categories 2 and 3 such that SBD diagnosis is not delayed. In a large retrospective study of 70,124 patients with positive FIT results, follow-up colonoscopies performed greater than 10 months after the positive FIT result were associated with a greater risk of colorectal cancer and more advanced disease at the time of diagnosis (OR1.48-2.25 and OR1.97-3.22, respectively). The extended waiting times for moderate to most urgent cases is not unique to our centre [21–23] and identifies a major gap in clinical practice where there is significant potential to improve coordination of patient care, improve patient outcomes while also saving substantial hospital and government expenditure. If we can identify patients presenting with symptoms who do not require colonoscopy, and manage them using more effective models of care, we will reduce the number of unnecessary invasive procedures and simultaneously reduce waiting times for individuals at highest risk of SBD.

Our study benefits from being a prospective analysis of a typical GP-referred population of patients. Specifically, we did not identify any significant demographic differences between the study population and those attending other CCC who were not recruited to the study.

There are some limitations to the study. Blood and stool tests were done at a pre-specified time point prior to colonoscopy and not at the time of the GP referral. In addition, the study does not include individuals with lower abdominal symptoms seen by their GP but not referred to a secondary care centre. Further studies in collaboration with primary healthcare centres will aim to address these points. Finally, our study is performed using a discovery cohort only. To ensure the robustness of our results, we have employed bootstrapping techniques; however to ensure generalisability the collection of a validation cohort is the next logical step.

Currently, there is limited application of evidence-based algorithms to target individuals at highest risk of SBD. Based on our data, incorporation of an SBD RAT early in the clinical pathway would be more clinically effective and safer for triaging symptomatic patients. The SBD RAT was specifically developed utilising blood and stool tests routinely requested in the primary health care setting. Compared to FIT alone, the addition of data from the lipid and chem20 blood panels to a FIT, a widely accepted test for colorectal cancer screening in the asymptomatic population, provided an increased sensitivity (24.5% vs. 11.6%) and specificity (96.5% vs. 95.9%) for detecting SBD. This new model generated markedly enhanced performance characteristics compared to the current symptoms-based model.

## Conclusion

It is critical that individuals with high risk of having significant bowel disease are triaged to the appropriate category with the shortest wait time. Here we provide a framework where evidence-based risk factors can be included in the diagnostic pathway for significant bowel pathology. To the best of our knowledge, this is the first study identifying a combination of blood markers along with FIT and demographic markers have a higher diagnostic accuracy for SBD than FIT alone.

## Abbreviations

BMI: Body mass index
BRS: Blood risk score
CCC: Colonoscopy consent clinic
CI: Confidence interval
FDR: False discovery rate
FIT: Faecal immunochemical test
GP: General practitioner
IBD: Inflammatory bowel disease
iFOB: Immunological faecal occult blood test
IQR: Interquartile range
NPV: Negative predictive value
OR: Odds ratio
PPV: Positive predictive value
RAT: Risk assessment tool
RBWH: Royal Brisbane and Women’s Hospital
SBD: Significant bowel disease
